# Only 1 in 10 patients achieve their rehabilitation goals at 1 year and 1 in 4 at 2 years following ACL reconstruction: An analysis of 907 patients

**DOI:** 10.1002/jeo2.70507

**Published:** 2025-11-03

**Authors:** Johan Högberg, Jakob Lindskog, Ramana Piussi, Rebecca Hamrin Senorski, Roland Thomeé, Kristian Samuelsson, Eric Hamrin Senorski

**Affiliations:** ^1^ Sportrehab Sportrehab Sports Medicine Clinic Gothenburg Sweden; ^2^ Sahlgrenska Sports Medicine Center Gothenburg Sweden; ^3^ Unit of Physiotherapy, Department of Health and Rehabilitation, Institute of Neuroscience and Physiology, Sahlgrenska Academy University of Gothenburg Gothenburg Sweden; ^4^ Department of Orthopaedics, Institute of Clinical Sciences, Sahlgrenska Academy University of Gothenburg Gothenburg Sweden; ^5^ Department of Orthopaedics Sahlgrenska University Hospital Mölndal Sweden

**Keywords:** ACL, anterior cruciate ligament, goal, patient‐reported outcomes, perceived knee function, rehabilitation

## Abstract

**Purpose:**

The aim of this study was to describe and compare demographic characteristics, perceived knee function and muscle function between patients who achieved their rehabilitation goals and those who did not at 1 and 2 years after anterior cruciate ligament (ACL) reconstruction.

**Methods:**

Data on patient demographics, results from patient‐reported outcomes (PROs), muscle strength and hop performance tests were extracted from Project ACL in April 2024. Patients treated with primary ACL reconstruction, ≥15 years of age at surgery, with complete data from PROs, muscle strength and hop performance tests at 1 or 2 years after surgery were included. The outcomes included comparisons of (1) demographics, (2) PROs and (3) muscle function tests between patients who achieved and those who did not achieve their rehabilitation goals at 1 and 2 years after ACL reconstruction. Alpha level was set at *p* < 0.05.

**Results:**

At 1 year, 762 patients were included of whom 100 patients (13%) achieved their rehabilitation goals. At 2 years, 145 patients were included, of whom 40 patients (28%) achieved their rehabilitation goals. Patients who achieved their rehabilitation goals at 1 year and 2 years reported less severe perceived knee symptoms (*d* = 0.24–0.46), greater perceived knee function (*d* = 0.52–0.77), greater knee related self‐efficacy (*d* = 0.42–0.68), more positive emotions, greater confidence and higher risk appraisal to return to sport (*d* = 0.47–0.82), and were active at a higher physical activity level (*p* < 0.001), despite lower set rehabilitation goals than patients who did not achieve their rehabilitation goals.

**Conclusions:**

Patients who achieve their rehabilitation goals after ACL reconstruction are characterized by higher perceived knee function, less severe knee symptoms, higher knee self‐efficacy, more positive emotions, stronger confidence and higher risk appraisal to return to sport and engage in higher activity levels compared to patients who did not achieve their rehabilitation goals.

**Level of Evidence:**

Level III, retrospective observational registry study.

**Abbreviations**
ACL: anterior cruciate ligament
ACLR: anterior cruciate ligament reconstruction
ACL‐RSI: anterior cruciate ligament–return to sport after injury scale
BMI: body mass index
CI: confidence interval
HT: hamstring tendon
ICC: intraclass coefficient
KOOS: Knee injury and Osteoarthritis Outcome Score
KOOS‐P: KOOS Pain
KOOS‐QoL: KOOS Quality of Life
KOOS‐S: KOOS Symptoms
KOOS‐SR: KOOS Sports and Recreation
K‐SES_18_
: 18‐item version of the Knee Self‐Efficacy Scale
LSI: limb symmetry index
n: numbers
n.s: non‐significant
PROs: patient‐reported outcomes
PT: patellar tendon
QT: quadriceps tendon
RECORD: REporting of studies Conducted using Observational Routinely Collected health Data
Tegner: Tegner Activity Scale

## BACKGROUND

The anterior cruciate ligament (ACL) injury represents a devastating injury for individuals engaged in pivoting activities [7]. Whether treatment after ACL injury/reconstruction is regarded as successful or not depends on the definition of success. Successful outcome after ACL injury/reconstruction based on an expert consensus [19] was characterized by minimal knee effusion, absence of knee instability, quadriceps and hamstring strength with a limb symmetry index (LSI) of ≥90%, and to achieve satisfactory subjective knee function and physical activity level assessed by patient‐reported outcomes (PROs) (specific PROs and threshold values were not agreed upon). While these criteria can serve as guiding metrics for clinicians, they may not entirely capture patient satisfaction with treatment outcome. Notably, there is a discrepancy between surgeons' and patients' rating of outcomes after ACL reconstruction, where surgeons rate outcomes more favourably than patients [22]. Surgeons are more likely to prioritize surgical‐related outcomes such as graft rupture, knee laxity and posttraumatic knee osteoarthritis, while patients consider return to daily activities, sport, and work as more important [20].

Outcomes after ACL reconstruction should be defined from the patient's perspective, emphasizing value‐based health care, that is, the measured improvement in a patient's health outcome [25]. Ardern et al. [2] reported that 44% of patients were happy or satisfied with their current knee function, with an average follow‐up of 3 years (ranging from 1 to 7 years) after ACL reconstruction. To employ strategies such as goal setting, may present a promising avenue for enhancing psychological outcomes, including satisfaction after ACL reconstruction [1]. A goal is defined as a specific objective or target that an individual endeavours to attain [18]. Given that less than half of patients report feeling happy or satisfied after ACL reconstruction [2], there is a need to delve further into which factors that distinguish patients who successfully achieve their rehabilitation goals and those who do not. This serves as an initial step towards improving patient satisfaction after an ACL injury.

The aim of this study was to describe and compare demographic characteristics, PROs, and muscle function between patients who achieved their rehabilitation goals and those who did not at 1 and 2 years after ACL reconstruction. The hypothesis was that patients who achieved their rehabilitation goals would be characterized by greater knee‐related self‐efficacy, more positive emotions, stronger confidence and higher risk appraisal to return to sport, as well as having greater symmetry for muscle strength and hop performance than patients who did not achieve their rehabilitation goals at 1 and 2 years after ACL reconstruction.

## METHODS

This study was designed as an explorative cross‐sectional study based on a local rehabilitation registry; Project ACL located in Gothenburg, Sweden. The REporting of studies Conducted using Observational Routinely Collected health Data (RECORD) statement was used as guidance for the outline of the study [5] (Supporting Information S1: Table 1). Ethical approval has been given from the Regional Ethical Review Board in Gothenburg (265‐13, T023‐17) and the Swedish Ethical Review Authority (2020‐02501).

### Setting: Project ACL—A local rehabilitation registry

Project ACL started 2014 with the main objective to improve outcomes for patients after ACL injury. The registry is open to all patients who have sustained an ACL injury, irrespective of treatment (non‐reconstructive or reconstructive) or time after injury or reconstruction. Informed consent is required upon registration and withdrawal from the registry is possible at any time. Patients who participate in the registry are invited to respond to validated PROs, perform muscle strength and hop performance assessments according to a predefined schedule with injury or reconstruction as baseline, and thereafter at 10 weeks, 4, 8, 12, 18, 24 and 60 months, and then every fifth year. Results of the assessments are entered into the registry database and shared with the patients and the treating therapist to guide the rehabilitation process. The registered data are used for research purposes with the objective of improving ACL injury management and rehabilitation strategies.

### Population

Patients registered in the rehabilitation registry, Project ACL, were eligible for inclusion in the present study.

Inclusion criteria were the following:

1. Treated with ACL reconstruction,
2. ≥15 years old at the time of ACL reconstruction, and
3. complete data of PROs, muscle strength and hop performance at 1 or 2 years after ACL reconstruction.

The following exclusion criteria were applied:

1. Had sustained two or more ACL injuries,
2. contralateral autograft harvest, and
3. did not specify graft choice.

### Rehabilitation goals

To evaluate whether patients achieved their rehabilitation goals, they were asked the following question: ‘Is the rehabilitation still ongoing?’ (Yes/no).

For patients who responded affirmatively, a subsequent inquiry was posed: ‘What are the goals of the current rehabilitation?’ with the following response options:

a. To return to a higher level of sport/physical activity than preinjury.
b. To return to preinjury level of sport/physical activity.
c. To return to another sport/physical activity at the same or higher level than preinjury.
d. To return to my preinjury sport/physical activity or another sport/physical activity at a lower level than preinjury.
e. To manage daily activities.
f. Other.

In cases where patients responded negatively to the question ‘Is the rehabilitation still ongoing?,’ they were subsequently asked: ‘Did you achieve your goals with your rehabilitation?’ (Yes/no), and ‘What was your goal of rehabilitation?’ with the same response options as above.

### Muscle function tests: Isokinetic muscle strength and hop performance tests

Preceding isokinetic muscle strength assessment was a 10‐min warm‐up session on a stationary bike at submaximal intensity. After the stationary bike warm‐up, an additional warm‐up and familiarization session was conducted with an isokinetic dynamometer (Biodex System 4; Biodex Medical System) [11]. Patients performed the isokinetic strength assessment while seated, initiating with a concentric knee extension followed immediately by a concentric knee flexion at an angular velocity of 90°/s and within a range of motion from 0 to 90° of knee flexion. Ten submaximal repetitions were performed at 50% of maximal effort, followed by another 10 submaximal repetitions at 75% of maximal effort. Preceding the maximal attempts, a submaximal repetition of 90% of maximal effort was performed. The peak torque measured in Newton metres (Nm) was recorded from 3 to 4 single repetitions trials, with a 40‐s rest interval between trials, and was entered into the registry database. The isokinetic concentric test mode of the Biodex has demonstrated excellent reliability for test–retest measures of single repetitions of peak torque with intraclass coefficient (ICC) values ranging from 0.95 to 0.97 [10, 11].

After muscle strength assessment in the Biodex, patients moved to three hop tests: the vertical hop, hop for distance and the 30‐second side hop test. Prior to the vertical hop and hop for distance tests, patients were given the opportunity for familiarization with 2 to 3 submaximal trials, while for the 30‐second side hop test, familiarization with 10 hops was allowed. To maintain standardization across hop tests, patients were instructed to keep their hands behind their backs for all trials. Patients commenced the vertical hop tests with 3 maximal attempts, with the time from take‐off to landing converted to centimeters (cm) using the Muscle Lab (Ergotest Technology). For the hop for distance, patients were allowed 3 to 5 maximal attempts, with the distance in cm measured from the toes at takeoff to the heel at a stable landing, ensuring no movement of the foot, release of hands behind the back, or support with the opposite foot touching the floor upon landing. The third hop test, the 30‐second side hop test, involved patients attempting to hop as many times as possible past 2 lines spaced 40 cm apart within a 30‐s period. The total number of hops completed without touching a line was recorded. The best results from each hop test were recorded in the Project ACL database. The hop tests used in Project ACL have exhibited high test–retest reliability in patients who have undergone ACL reconstruction, with corresponding to ICC values ranging from 0.85 to 0.97 [12].

### Patient‐reported outcomes

The Knee Injury and Osteoarthritis Outcome Score (KOOS) comprise 5 subscales with a total of 42 items: pain (KOOS‐P), symptoms (KOOS‐S), function in daily living (KOOS‐ADL), function in sport and recreation (KOOS‐SR) and knee‐related quality of life (KOOS‐QoL). Each item within the scale is assessed using a 5‐point Likert Scale, which ranges from 0 to 4. The score within each subscale is then converted to a 0–100 scale. A score of 0 on each subscale indicates severe symptoms, while a score of 100 indicates the absence of symptoms. In Project ACL, the KOOS‐ADL has been removed due to deemed irrelevant by patients [27]. The KOOS has demonstrated acceptable reliability for test–retest measurements and responsiveness as an assessment tool across various knee injuries [23].

The 18‐item version of Knee Self‐Efficacy Scale (K‐SES_18_) demonstrates acceptable construct validity and acceptable test–retest reliability (ICC = 0.92) to evaluate knee‐related self‐efficacy in patients with ACL injury and reconstruction [4]. The K‐SES_18_ comprises two subscales: present knee self‐efficacy, encompassing 14 items, and future knee self‐efficacy, which comprise 4 items. Each item is assessed with an 11‐point Likert scale ranging from 0 to 10. Subsequently, mean scores are computed for each subscale, where 0 represent the lowest attainable knee self‐efficacy and 10 represent the highest achievable knee self‐efficacy.

The Anterior Cruciate Ligament Return To Sport after Injury scale (ACL‐RSI) is designed to assess the emotions, confidence and risk appraisal for patients to return to sport after ACL reconstruction [28]. The original scale comprises 12 items, with each item scored on an 11‐point Likert scale ranging from 0 to 10, whereas a modified version is used in Project ACL, ranging from 1 to 10. A score of 1 signifies an exceedingly negative psychological response, while a score of 10 denotes extremely positive emotions, confidence and risk appraisal on return to sport. The scale's scoring system has been adjusted in Project ACL, so that the minimum is 10 and the maximum achievable score is 100, as opposed to the original range of 0 to 100. The summarized score of the 12 items is superimposed to a 10–100 scale by dividing the score with 1.2. The scale has demonstrated acceptable divergent validity and internal consistency [28].

To estimate the level of physical activity, the Tegner Activity scale (Tegner) was used. The Tegner is designed to evaluate the extent of knee‐demanding activities, graded on a scale from 0 to 10, where higher scores indicate greater physical demands on the knee [24]. In Project ACL, a modified version of the Tegner is employed, spanning from 1 to 10. For example, a score of 1 corresponds to walking on even ground, while a score of 10 signifies engagement in elite‐level football. The Tegner has demonstrated acceptable test–test reliability, corresponding to an ICC value of 0.8, along with acceptable floor and ceiling effects [6]. In the present study, the preinjury Tegner and the present Tegner were used.

### Outcomes of interest

Data on patient demographics, results from PROs, muscle strength, and hop performance was extracted from Project ACL in April 2024. The outcomes of the present study were the comparisons in (1) demographics, (2) PROs, and (3) muscle function between patients who did and did not achieve their rehabilitation goals at 1 and 2 years after ACL reconstruction.

1. Demographics constituted sex (female/male), age (at ACL reconstruction), body mass index (BMI), days between ACL injury and ACL reconstruction, and graft choice (e.g., hamstring tendon or patellar tendon autograft).
2. PROs constituted responses to the KOOS subscales, K‐SES_18_, ACL‐RSI and Tegner.
3. Muscle function constituted results of the muscle strength and hop performance assessments, presented as the limb symmetry index (LSI), that is, results of the reconstructed leg divided with the nonreconstructed leg expressed as a percentage), and as relative muscle strength of the reconstructed leg (muscle strength divided by weight in kilograms).

### Statistical analysis

Descriptive statistics constituted mean with standard deviations, median with the 25th and the 75th percentiles, and count with percentages. Comparisons were made with independent *t*‐test for continuous data, Mann–Whitney *U*‐test for ordinal data, and Fisher's exact test for categorical data. In the event of a significant finding, defined as *p* < 0.05, the effect size (Cohen's *d*) with 95% confidence interval was calculated for parametric data with the reference values: small = 0.2–0.49, moderate = 0.5–0.79, and large = ≥0.8 [8]. Sensitivity analyses for patient demographics (sex, age, height and weight) were performed between patients with complete and incomplete data for muscle function tests and PROs at 1 and 2 years to address potential attrition bias. Significant differences with ≥0.2 in Cohen's *d* were considered to have a meaningful effect on our results. Statistical analyses were performed with Statistical Product and Service Solutions (IBM Corp. Released 2017. IBM SPSS Statistics for Windows, Version 29.0; IBM Corp.).

## RESULTS

**Figure 1:**
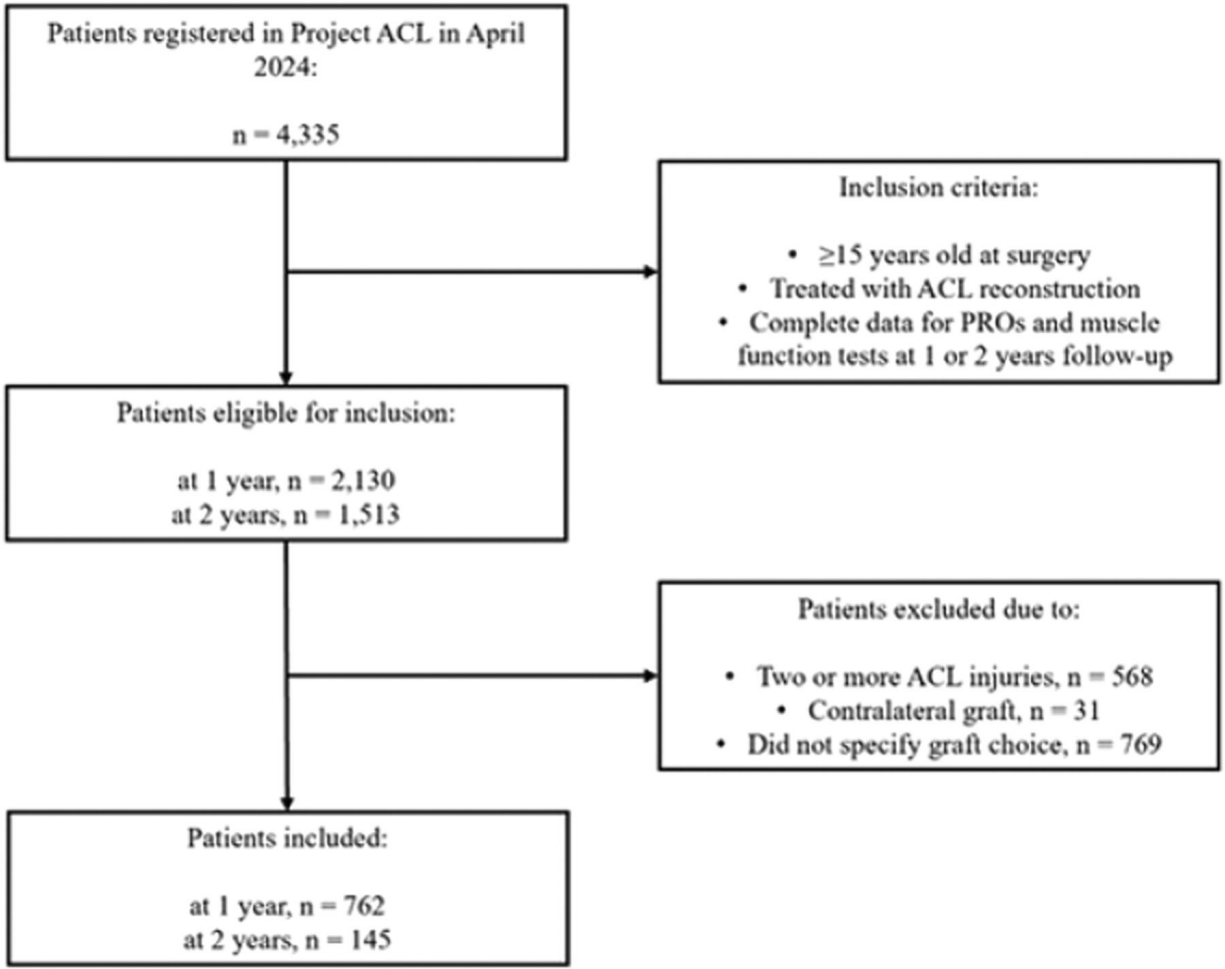
Flowchart of the selection process. ACL, anterior cruciate ligament; *n*, number; PROs, patient‐reported outcomes.

In April 2024, a total of 4,335 patients were registered in the rehabilitation registry Project ACL. Of these registered patients, 2,130 and 1,513 were deemed eligible for inclusion at 1 and 2 years after ACL reconstruction respectively. After application of exclusion criteria, 762 patients were included in the 1‐year follow‐up, and 145 were included in the 2‐year follow‐up (Figure 1).

### 1 year

**Figure 2:**
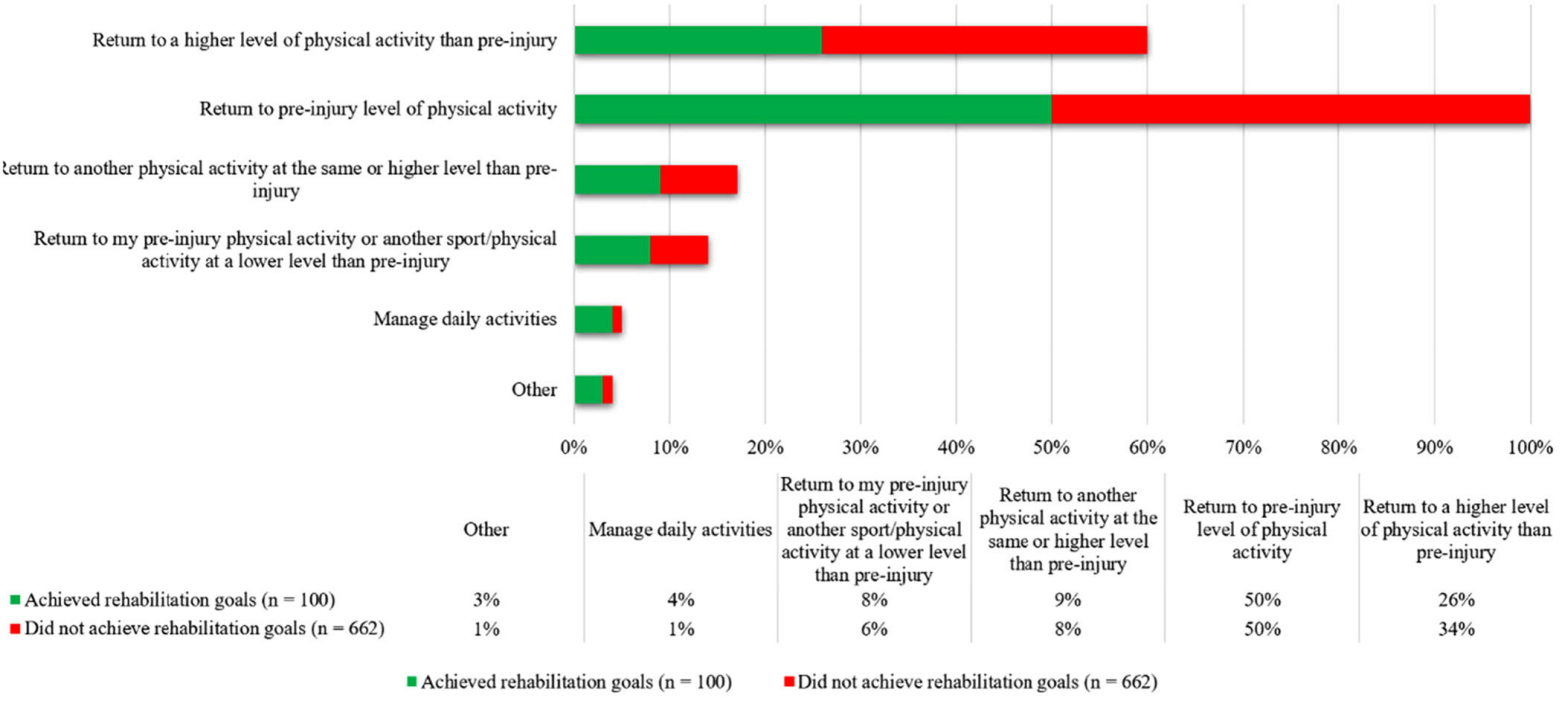
Rehabilitation goals for patients included 1 year after ACL reconstruction. Fisher's exact test demonstrated a significant difference in rehabilitation goals between groups (*p* = 0.04). *n*, number.

At 1 year after ACL reconstruction, 100 patients (13%) reported that they had achieved their rehabilitation goals. There was a significant difference in rehabilitation goals between patients who did and those who did not achieve their rehabilitation goals by 1 year after surgery (*p* = 0.04). Twenty‐six (26%) of patients who achieved their rehabilitation goals aimed to return to a higher level of physical activity than preinjury, compared to 228 (34%) of patients who did not achieve their rehabilitation goals at 1 year after surgery (Figure 2).

Patients who achieved their rehabilitation goals 1 year after ACL reconstruction were more often reconstructed with hamstring tendon autograft (89% vs. 78.9%) than with patellar tendon autograft (8% vs. 19.8%) compared to patients who did not achieve rehabilitation goals (*p* = 0.004). Additionally, 80% of patients who achieved their rehabilitation goals reported to perform pivoting activities preinjury (Tegner 6–10), compared to 87.6% of patients who did not achieve their goals (*p* = 0.032). Patients who achieved their rehabilitation goals were active at a higher activity level 1 year after ACL reconstruction compared to patients who did not achieve rehabilitation goals (*p* < 0.001). At 1 year, 77% of patients who achieved their rehabilitation goals were active with pivoting activities (Tegner 6–10), whereas 54.7% of patients who did not achieve their rehabilitation goals performed pivoting activities (Table 1).

**Table 1:** Demographics between groups 1 year after ACL reconstruction.

	Achieved rehabilitation goals (*n* = 100)	Did not achieve rehabilitation goals (*n* = 662)	*p* value
Female, *n* (%)	50 (50%)	352 (53%)	0.59
Age at ACLR, years	27 ± 10	26 ± 9	0.39
Days between injury and ACLR, median with 25th and 75th percentiles	143 (71–320)	134 (83–260)	0.38
BMI, kg/m^2^	24 ± 2	24 ± 3	0.88
Graft choice, *n* (%)			0.004
HT	89 (89.0%)	522 (78.9%)	
PT	8 (8.0%)	131 (19.8%)	
Allograft	0 (0%)	5 (0.8%)	
QT	1 (1.0%)	1 (0.2%)	
Other	2 (2.0%)	3 (0.5%)	
Preinjury Tegner, median with 25th and 75th percentiles	8 (7–9)	8 (7–9)	0.032
1, n (%)	1 (1.0%)	4 (0.6%)	
2, n (%)	2 (2.0%)	5 (0.8%)	
3, n (%)	6 (6.0%)	19 (2.9%)	
4, n (%)	3 (3.0%)	27 (4.1%)	
5, n (%)	8 (8.0%)	27 (4.1%)	
6, n (%)	4 (4.0%)	58 (8.8%)	
7, n (%)	23 (23.0%)	98 (14.8%)	
8, n (%)	24 (24.0%)	154 (23.3%)	
9, n (%)	22 (22.0%)	187 (28.2%)	
10, n (%)	7 (7.0%)	83 (12.5%)	
Present Tegner, median with 25th and 75th percentiles	7 (6–8)	6 (4–7)	<0.001
1, n (%)	0 (0%)	3 (0.5%)	
2, n (%)	3 (3.0%)	17 (2.6%)	
3, n (%)	0 (0%)	81 (12.2%)	
4, n (%)	13 (13.0%)	125 (18.9%)	
5, n (%)	7 (7.0%)	74 (11.2%)	
6, n (%)	14 (14.0%)	82 (12.4%)	
7, n (%)	23 (23.0%)	126 (19.0%)	
8, n (%)	17 (17.0%)	71 (10.7%)	
9, n (%)	19 (19.0%)	64 (9.7%)	
10, n (%)	4 (4.0%)	19 (2.9%)	

Abbreviations: ACLR, anterior cruciate ligament reconstruction; BMI, body mass index; HT, Hamstring Tendon; *n*, number; PT, patellar tendon; QT, quadriceps tendon; Tegner, Tegner Activity Scale.

Patients who achieved their rehabilitation goals 1 year after ACL reconstruction reported less severe symptoms and greater function according to the KOOS subscales, as well as a higher knee self‐efficacy at the present (*p* < 0.001, *d* = 0.52, 95% CI [0.31; 0.74]), for the future (*p* < 0.001, *d* = 0.42, 95% CI [0.21; 0.74]), and had more positive emotions, stronger confidence and greater risk appraisal to return to sport (*p* < 0.001, *d* =0.47 95% CI [0.25; 0.70]) compared to patients who did not achieve their rehabilitation goals (Table 2).

**Table 2:** Patient‐reported outcomes and muscle function tests between groups 1 year after ACL reconstruction.

	Achieved rehabilitation goals (*n* = 100)	Did not achieve rehabilitation goals (*n* = 662)	*p* value	Cohens' *d* with 95% CI
KOOS‐P	92 ± 10	88 ± 10	0.01	0.35 (0.13; 0.56)
KOOS‐S	83 ± 14	79 ± 15	0.03	0.24 (0.03; 0.45)
KOOS‐SR	83 ± 14	74 ± 19	<0.001	0.52 (0.31; 0.74)
KOOS‐QoL	72 ± 16	60 ± 17	<0.001	0.70 (0.49; 0.92)
K‐SES_18_ Present	9.1 ± 0.9	8.5 ± 1.2	<0.001	0.52 (0.31; 0.74)
K‐SES_18_ Future	8.2 ± 1.8	7.5 ± 1.7	<0.001	0.42 (0.21; 0.63)
ACL‐RSI	71 ± 22	61 ± 20	<0.001	0.47 (0.25; 0.70)
Quadriceps LSI (%)	96% ± 9.9%	95% ± 11%	0.11	
Relative quadriceps strength, Nm/kg	2.7 ± 0.5	2.7 ± 0.5	0.61	
Hamstring LSI (%)	98% ± 13%	98% ± 13%	0.89	
Relative hamstring strength, Nm/kg	1.5 ± 0.3	1.5 ± 0.3	0.56	
Vertical hop LSI (%)	92% ± 12%	89% ± 16%	0.07	
Hop for distance LSI (%)	96% ± 9.1%	94% ± 10%	0.05	
30‐second side hop LSI (%)	101% ± 15%	95% ± 17%	0.004	0.31 (0.10; 0.52)

Abbreviations: ACL‐RSI, anterior cruciate ligament–return to sport after injury scale; CI, confidence interval; KOOS, Knee injury and Osteoarthritis Outcome Score; KOOS‐P, KOOS Pain; KOOS‐S, KOOS Symptoms; KOOS‐SR, KOOS Sports and Recreation; KOOS‐QoL, KOOS Quality of Life; K‐SES_18_, 18‐item version of the Knee Self‐Efficacy Scale; LSI, Limb Symmetry Index; n, number.

Patients who achieved their rehabilitation goals had greater LSI in the 30‐second side hop test compared to patients who did not (*p* = 0.004, *d* = 0.31 95% CI [0.10; 0.52]) (Table 2).

#### Sensitivity analysis

There were significant differences in age at ACL reconstruction (26 vs. 28 years) and weight (72.5 vs. 73.7 kg) between patients with complete and incomplete data 1 year after ACL reconstruction, where patients with complete data were younger and had a greater weight. However, the effect sizes were negligible (*d* < 0.2) (Supporting Information S1: Table 2).

### 2 years

**Figure 3:**
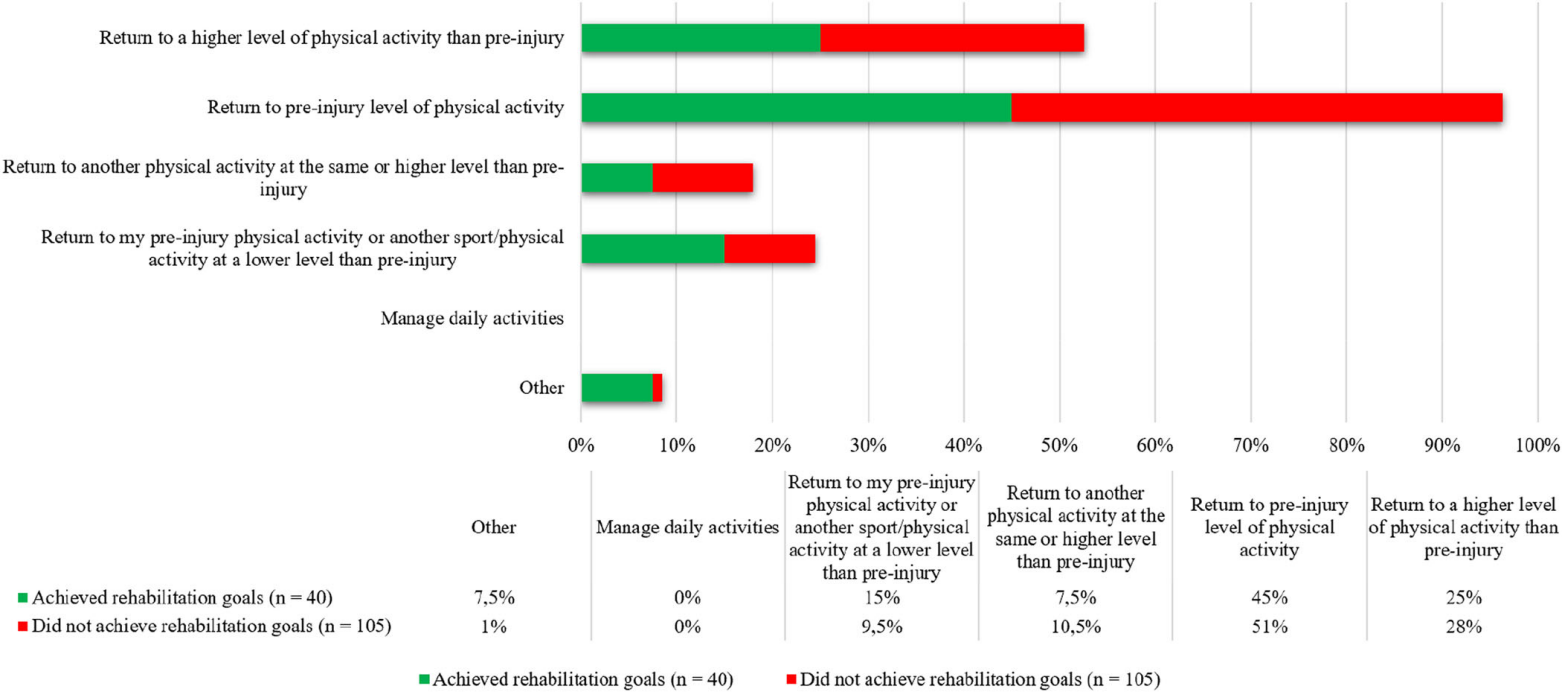
Rehabilitation goals for patients included 2 years after ACL reconstruction. Fisher's exact test demonstrated a non‐significant difference in rehabilitation goals between groups (*p* = 0.25). ACL, anterior cruciate ligament; n, number.

Two years after ACL reconstruction 40 patients (28%) reported they had achieved their rehabilitation goals. There were no significant differences in rehabilitation goals between patients who achieved their goals with patients who did not (*p* = 0.25) (Figure 3).

At 2 years, 87.5% of patients who achieved their rehabilitation goals were active with pivoting activities (Tegner 6–10), whereas 54.4% of patients who did not achieve their rehabilitation goals performed pivoting activities (*p* < 0.001) (Table 3).

**Table 3:** Demographics between groups 2 years after ACL reconstruction.

	Achieved rehabilitation goals (*n* = 40)	Did not achieve rehabilitation goals (*n* = 105)	*p* value
Female, *n* (%)	19 (48%)	44 (42%)	0.58
Age at ACLR, years	26 ± 10	29 ± 10	0.12
Days between injury and ACLR, median with 25th and 75th percentiles	111 (75–244)	142 (85–315)	0.29
BMI, kg/m^2^	24 ± 2	24 ± 3	0.17
Graft choice, *n* (%)			0.84
HT	36 (90.0%)	90 (86%)	
PT	4 (10.0%)	14 (13.3%)	
Allograft	0 (0%)	1 (0.7%)	
QT	0 (0%)	0 (0%)	
Other	0 (0%)	0 (0%)	
Preinjury Tegner, median with 25th and 75th percentiles	8 (7–9)	7 (6–9)	0.36
1, n (%)	0 (0%)	0 (0%)	
2, n (%)	0 (0%)	2 (1.9%)	
3, n (%)	2 (5.0%)	5 (4.8%)	
4, n (%)	2 (5.0%)	8 (7.6%)	
5, n (%)	1 (2.5%)	7 (6.7%)	
6, n (%)	2 (5.0%)	16 (15.2%)	
7, n (%)	7 (17.5%)	16 (15.2%)	
8, n (%)	9 (22.5%)	15 (14.3%)	
9, n (%)	12 (30.0%)	24 (22.9%)	
10, n (%)	5 (12.5%)	12 (11.4%)	
Present Tegner, median with 25th and 75th percentiles	7 (6–9)	6 (4–7)	<0.001
1, n (%)	0 (0%)	2 (1.9%)	
2, n (%)	0 (0%)	2 (1.9%)	
3, n (%)	1 (2.5%)	12 (11.4%)	
4, n (%)	1 (2.5%)	19 (18.1%)	
5, n (%)	3 (7.5%)	13 (12.4%)	
6, n (%)	7 (17.5%)	19 (18.1%)	
7, n (%)	9 (22.5%)	17 (16.2%)	
8, n (%)	6 (15.0%)	9 (8.6%)	
9, n (%)	9 (22.5%)	9 (8.6%)	
10, n (%)	4 (10.0%)	3 (2.9%)	

Abbreviations: ACLR, anterior cruciate ligament reconstruction; BMI, body mass index; HT, Hamstring Tendon; *n*, number; PT, Patellar Tendon; Tegner, QT, Quadriceps Tendon; Tegner Activity Scale;.

Patients who achieved their rehabilitation goals 2 years after ACL reconstruction had less severe symptoms and greater function according to the KOOS subscales, as well as a higher knee self‐efficacy both at the present (*p* < 0.001, *d* = 0.68 95% CI [0.31; 1.05]) and for the future (*p* < 0.001, *d* = 0.44 95% CI [0.07; 0.81]), compared to patients who did not achieve their rehabilitation goals. Patients who achieved their rehabilitation goals also had more positive emotions, greater confidence and higher risk appraisal to return to sport compared to patients who did not achieve their rehabilitation goals (*p* < 0.001, *d* = 0.82 95% CI [0.43; 1.21]). Patients who achieved their rehabilitation goals had a greater LSI in the hop for distance test compared to patients who did not (*p* < 0.001, *d* = 0.65 95% CI [0.27; 1.02]) (Table 4).

**Table 4:** Patient‐reported outcomes and muscle function tests between groups 2 years after ACL reconstruction.

	Achieved rehabilitation goals (*n* = 40)	Did not achieve rehabilitation goals (*n* = 105)	*p* value	Cohen's *d* with 95% CI
KOOS‐P	92 ± 10	88 ± 10	0.02	0.46 (0.09; 0.82)
KOOS‐S	85 ± 14	79 ± 15	0.02	0.43 (0.06; 0.79)
KOOS‐SR	84 ± 17	71 ± 20	<0.001	0.67 (0.29; 1.04)
KOOS‐QoL	74 ± 18	61 ± 17	<0.001	0.77 (0.39; 1.14)
K‐SES_18_ Present	9.2 ± 0.9	8.4 ± 1.3	<0.001	0.68 (0.31; 1.05)
K‐SES_18_ Future	7.5 ± 1.8	6.5 ± 2.3	0.02	0.44 (0.07; 0.81)
ACL‐RSI	74 ± 19	56 ± 22	<0.001	0.82 (0.43; 1.21)
Quadriceps LSI (%)	99% ± 9.2%	98% ± 10%	0.55	
Relative quadriceps strength, Nm/kg	2.8 ± 0.5	2.7 ± 0.4	0.13	
Hamstring LSI (%)	102% ± 15%	100% ± 18%	0.52	
Relative hamstring strength, Nm/kg	1.6 ± 0.3	1.5 ± 0.3	0.31	
Vertical hop LSI (%)	95% ± 12%	91% ± 13%	0.10	
Hop for distance LSI (%)	100% ± 6.7%	94% ± 9.3%	<0.001	0.65 (0.27; 1.02)
30‐second side hop LSI (%)	95% ± 14%	94% ± 17%	0.66	

Abbreviations: ACL‐RSI, anterior cruciate ligament–return to sport after injury scale; CI, confidence interval; KOOS, Knee injury and Osteoarthritis Outcome Score; KOOS‐P, KOOS Pain; KOOS‐S, KOOS Symptoms; KOOS‐SR, KOOS Sports and Recreational; KOOS‐QoL, KOOS Quality of Life; K‐SES_18_, 18‐item version of the Knee Self‐Efficacy Scale; LSI, Limb Symmetry Index; *n*, number.

#### Sensitivity analysis

There were significant differences in the proportion of women (43.4% vs. 52.1%), age at ACL reconstruction (29 vs. 27 years) and height (176.0 vs. 174.4 kg) between patients with complete and incomplete data 2 years after ACL reconstruction. Patients with complete data had a greater proportion of men, were older and taller. However, the effect sizes for age and height were negligible (*d* < 0.2) (Supporting Information S1: Table 3).

## DISCUSSION

One in 10 patients and 1 in 4 achieved their rehabilitation goals at 1 year and 2 years after ACL reconstruction, respectively. Patients who achieved their rehabilitation goals at 1 year were to a greater extent treated with a hamstring tendon autograft, had less severe perceived knee symptoms, greater perceived knee function, greater knee‐self‐efficacy, more positive emotions, greater confidence and higher risk appraisal to return to sport, and were active at a higher activity level, despite having lower set physical demanding rehabilitation goals than patients who did not achieve their rehabilitation goals.

Similarly, patients who achieved their rehabilitation goals at 2 years reported less severe perceived knee symptoms, greater perceived knee function and knee‐self‐efficacy, had more positive emotions, greater confidence and higher risk appraisal to return to sport, and participated in higher activity levels compared to patients who did not achieve their rehabilitation goals.

### Perceived knee function and activity level

More patients who did not achieve rehabilitation goals aimed to return to a higher physical activity level than preinjury (34.4% vs. 26.0%) at 1 year after ACL reconstruction. A greater proportion of these patients (87.6% vs. 80%) also participated in high knee‐demanding activities preinjury (Tegner 6–10). However, patients who achieved their rehabilitation goals reported higher current physical activity levels. The proportion aiming to return to preinjury physical activity levels was identical (50% in both groups), suggesting that an aim to return to a greater activity level than preinjury at 1 year may be unrealistic for most patients after ACL reconstruction, and to aim for return to preinjury physical level or lower may be a more realistic goal.

Patients who achieved their rehabilitation goals reported lower perceived knee pain and symptoms, greater perceived function in sports and recreation, as well as a greater quality of life measured with the KOOS at both 1 and 2 years, with small to moderate effect (*d* = 0.24–0.77). In line with our findings, Webster et al. [29] reported that patients who had less severe subjective knee symptoms and a greater subjective knee function returned to their preinjury level of sports to a higher degree. In addition, a systematic review [30] reported that patients who returned to sport after ACL reconstruction had a higher knee self‐efficacy and more positive emotions, stronger confidence and higher risk appraisal to return to sport compared to patients who did not, despite similar knee function scores. Thus, patients who achieve rehabilitation goals, often defined as return to preinjury activity (but not always), report higher perceived knee function and knee self‐efficacy, more positive emotions, stronger confidence and higher risk appraisal to return to sport than patients who do not achieve their rehabilitation goals.

While this study cannot determine causality due to the cross‐sectional design, it is possible that achieving rehabilitation goals contributes to better outcomes. Conversely, whether targeted psychological interventions could improve rehabilitation goal achievement remains to be investigated.

### Muscle function

At 1 year after ACL reconstruction, a small but statistically significant difference in LSI for the 30‐second side hop test was observed between the groups (101% vs. 95%). No significant differences were observed in quadriceps or hamstring strength, whether assessed as LSI or relative strength, between patients who achieved their rehabilitation goals with those who did not. Given the small effect size, the side hop difference may be a false positive and lacks clinical relevance.

At 2 years after ACL reconstruction, patients who achieved rehabilitation goals had significantly greater hop for distance LSI than patients who did not (100% vs. 94%) with a moderate effect size. However, this test has been criticized for allowing compensatory movement patterns, potentially masking knee function with greater contribution from the hip and ankle [16]. To merely evaluate the symmetry in the hop distance between the limbs may therefore provide insufficient information of the patients' actual knee function in the reconstructed limb [15]. In addition, psychological factors such as a lower preoperative knee self‐efficacy [26], less positive emotions, lower confidence and risk appraisal [9] and greater kinesiophobia [3] have been associated with poorer hop performance and more severe perceived knee symptoms [13].

Overall, no meaningful differences in muscle strength (relative or LSI) were observed between groups at either time point. Whether the observed hop distance LSI difference stems from physical or psychological factors [3, 9, 26] remains unclear.

### Graft choice

More patients who achieved rehabilitation goals at 1 year after ACL reconstruction received hamstring tendon autografts (89% vs. 79.8%), while those who did not were more often treated with patellar tendon autograft (19.8% vs. 8%). Patients reconstructed with hamstring tendon autografts have been associated with greater likelihood of achieving the minimal important change in KOOS‐SR and KOOS‐QoL at 1 year after ACL reconstruction [14], possibly due to lower rates of anterior knee pain compared to patients reconstructed with patellar tendon autografts [21]. However, at a minimum of 2 years, no difference in PROs can be found between the respective graft choices [31]. Thus, 1 year of rehabilitation may not be sufficient for most patients treated with patellar tendon autografts after ACL reconstruction to achieve their rehabilitation goals if their goal is to return to a higher level of physical activity than preinjury.

### Limitations

There are some limitations to this study. With a cross‐sectional study design, only observed differences between groups in the variables analyzed can be reported, not what causes or predicts the achievement of rehabilitation goals. We performed several analyses, which increases the risk for false positive findings. To address the risk of type I errors, we complemented the comparative analyses with effect sizes to assess the magnitude of significant findings. To evaluate patients' rehabilitation goals, they were posed with predefined answers which may not be in line with patients' rehabilitation goals. However, patients had the opportunity to select the response ‘other’ and write their goal in free text, which only 1% and 3% did at 1 and 2 years, respectively. Patients with ACL injury may choose to participate in Project ACL that forms the basis of the present study. As part of their participation, standardized muscle function tests and validated PROs are provided continuously throughout the rehabilitation. However, it is important to note that the rehabilitation process for each patient is individualized and managed by their own physical therapist, rather than by Project ACL. Data on concomitant injuries is not available in Project ACL, which can be a potential confounder in our analysis. Concomitant injuries, such as meniscus and cartilage damage may result in worse perceived knee symptoms [17], and may negatively affect the achievement of rehabilitation goals. The KOOS has demonstrated acceptable reliability for test–retest measurements and responsiveness as an assessment tool across various knee injuries, however, KOOS is not specifically validated or reliable for patients with ACL injuries [23]. Despite this, KOOS is commonly used within ACL literature. Furthermore, 1,479 and 2,073 patients registered in Project ACL did not have complete data of demographics, PROs and muscle function at 1 year and 2 years, respectively. This increases the risk of attrition bias. A sensitivity analysis was performed to address the potential attrition bias which resulted in some differences with effect sizes below 0.2. Nevertheless, there were fewer women that had complete data than men at 2 years (∆ = 8.7%). This difference increases the risk of attrition bias for our analyses at 2 years after surgery and induces caution in the generalization of the results and highlights the need for future studies with more complete follow‐up data to confirm our findings.

## CONCLUSION

Patients who achieve their rehabilitation goals after ACL reconstruction are characterized by higher perceived knee function, less severe knee symptoms, higher knee self‐efficacy, more positive emotions, stronger confidence and higher risk appraisal to return to sport and engage in higher activity levels compared to patients who did not achieve their rehabilitation goals. Notably, patients who achieved rehabilitation goals at 1 year had lower knee‐demanding rehabilitation goals compared to patients who did not achieve their goals. Thus, aiming to return to a higher physical activity level than preinjury may not be realistic for most patients at 1 year after ACL reconstruction.

## AUTHOR CONTRIBUTIONS

Johan Högberg draughted the initial version of the manuscript. Johan Högberg and Eric Hamrin Senorski contributed substantially to the acquisition of the data and the analysis of the data, and they are responsible for draughting the manuscript and revising it critically for important intellectual content. Jakob Lindskog, Ramana Piussi, Rebecca Hamrin Senorski, Roland Thomeé, and Kristian Samuelsson made large contributions to the revision and design of the work. Johan Högberg and Eric Hamrin Senorski are responsible for the concept of design.

## CONFLICT OF INTEREST STATEMENT

K.S. is a board member of Getinge A.B.

## ETHICS STATEMENT

All patients received written and oral information of the project gave informed consent. The study was approved by the Swedish Ethical Review Authority (2020‐02501).

## Supporting information

Supplementary files.

## Data Availability

Data is available upon reasonable request.
